# Validity of tissue homogeneity in confocal laser endomicroscopy on the diagnosis of laryngeal and hypopharyngeal squamous cell carcinoma

**DOI:** 10.1007/s00405-022-07304-y

**Published:** 2022-02-28

**Authors:** Matti Sievert, Marc Aubreville, Antoniu-Oreste Gostian, Konstantinos Mantsopoulos, Michael Koch, Sarina Katrin Mueller, Markus Eckstein, Robin Rupp, Florian Stelzle, Nicolai Oetter, Andreas Maier, Heinrich Iro, Miguel Goncalves

**Affiliations:** 1 Department of Otorhinolaryngology, Head and Neck Surgery, Friedrich-Alexander-Universität Erlangen-Nürnberg, University Hospital, Waldstrasse 1, 91054 Erlangen, Germany; 2 Technische Hochschule Ingolstadt, Ingolstadt, Germany; 3 Institute of Pathology, Friedrich-Alexander-Universität Erlangen-Nürnberg, University Hospital, Erlangen, Germany; 4 Department of Maxillofacial Surgery, Friedrich-Alexander-Universität Erlangen-Nürnberg, University Hospital, Erlangen, Germany; 5 Pattern Recognition Laboratory, Computer Science, Friedrich-Alexander-Universität Erlangen-Nürnberg, Erlangen, Germany; 6 Department of Otorhinolaryngology, Plastic Head and Neck Surgery, Rheinische Westfälische Technische Hochschule Aachen, University Hospital, Aachen, Germany

**Keywords:** Confocal laser endomicroscopy, Head and neck cancer, Classification system, Non-invasive histological imaging, Larynx, Pharynx

## Abstract

**Purpose:**

Confocal laser endomicroscopy (CLE) allows imaging of the laryngeal mucosa in a thousand-fold magnification. This study analyzes differences in tissue homogeneity between healthy mucosa and squamous cell carcinoma (SCC) via CLE.

**Materials and methods:**

We included five SCC patients with planned total laryngectomy in this study between October 2020 and February 2021. We captured CLE scans of the tumor and healthy mucosa. Analysis of image homogeneity to diagnose SCC was performed by measuring the signal intensity in four regions of interest (ROI) in each frame in a total of 60 sequences. Each sequence was assigned to the corresponding histological pattern, derived from hematoxylin and eosin staining. In addition, we recorded the subjective evaluation of seven investigators regarding tissue homogeneity.

**Results:**

Out of 3600 images, 1620 (45%) correlated with benign mucosa and 1980 (55%) with SCC. ROIs of benign mucosa and SCC had a mean and standard deviation (SD) of signal intensity of, respectively, 232.1 ± 3.34 and 467.3 ± 9.72 (*P* < 0.001). The mean SD between the four different ROIs was 39.1 ± 1.03 for benign and 101.5 ± 2.6 for SCC frames (*P* < 0.001). In addition, homogeneity yielded a sensitivity and specificity of 81.8% and 86.2%, respectively, regarding the investigator-dependent analysis.

**Conclusions:**

SCC shows a significant tissue inhomogeneity in comparison to the healthy epithelium. The results support this feature’s importance in identifying malignant mucosa areas during CLE examination. However, the examiner-dependent evaluation emphasizes that homogeneity is a sub-criterion that must be considered in a broad context.

## Introduction

Squamous cell carcinoma (SCC) is considered responsible for over 90% of all pharyngeal and nearly 100% of the larynx malignancies [1]. In the diagnostic process, microscopical, histopathological assessment of tissue biopsy is currently considered the gold standard. Various non-invasive optical imaging methods have emerged as potential alternatives to invasive tissue biopsy, albeit mostly in experimental settings. The most promising are confocal laser endomicroscopy (CLE) and optical coherence tomography [2–5]. Probe-based CLE enables the visualization of live mucosal tissue at a cellular level and has been particularly established in mucosal diagnostics of the gastrointestinal tract in the last decade [6]. CLE has been broadly studied in identification of dysplasia in Barret’s esophagus, gastric and colorectal polyps [7, 8]. Furthermore, it has been shown to be beneficial due to its dynamic in-vivo examination in the assessment of disease activity and development of dysplasia in inflammatory bowel disease and celiac disease [9, 10]. After initially being intensively studied in gastroenterology, research has expanded to other areas, such as pneumology [11], urology [12], neurosurgery [13], and the head and neck region. This method utilizes a small laser scanning probe (1–3 mm) gently applied to the area of interest. A field of view of up to 600 μm enables a magnification up to 1000 times and the real-time visualization of the superficial tissue architecture, with a defined focus depth of usually 60–350 μm [14]. Using fluorescein to outline the intercellular spaces and visualization of blood vessels [6], CLE provides “real-time” optical biopsies [15]. The images acquired by probe-based CLE reassemble histopathological tangential sections of the mucosa at the defined depth given by the probe. The reliable interpretation and classification in malignant and normal findings are not trivial, require training, and are usually aided by classification systems [16–19]. In these classification systems, capillary aberrations and tissue homogeneity play a significant role. Even though an 80–90% accuracy is reported in earlier publications, examiner dependency is still a major problem to broad implementation of this technique [20–22].

Tissue inhomogeneity in SCC is a component of several classification systems [16–19]. In most cases, this feature appears to be reliable in identifying malignancy; however, to the best of our knowledge, an objective analysis of this parameter did not take place yet. We hypothesize that malignant tissue shows, even in the small area investigated at any given point by probe-based CLE of 240 μm, a quantifiable regional variation that correlates to histology and is significantly different from the healthy epithelium. This study aimed to objectively—examiner independent—assess the differences in tissue homogeneity between healthy epithelium and SCC in CLE compared to examiner dependent analyses related to histopathological findings.

## Materials and methods

### Study design

This prospective pilot study was conducted at a tertiary hospital and academic cancer center (Department of otorhinolaryngology, head and neck surgery, Friedrich-Alexander-University of Erlangen-Nuremberg, Erlangen, Germany). The study was approved by the local institutional ethics committee (approval number 60_14 B) and carried out following the declaration of Helsinki. In addition, we obtained written informed consent from all study participants.

### Eligibility criteria

A total of five consecutive patients with confirmed SCC of the pharynx or larynx were included in this study. Exclusion criteria were a prior treatment of any head and neck cancer, distant metastasis, radiotherapy in the head and neck area, pregnancy, thyroid dysfunction, minority, severe kidney failure, and allergy to fluorescein.

### Confocal laser endomicroscopy system and data acquisition

We performed intraoperative imaging using a GastroFlex probe and a 488 nm CellvizioTM laser scanning system (Mauna Technologies, Paris, France). The probe has a diameter of 2.6 mm, a penetration depth of 55–65 µm, a field of view of 240 µm, and a resolution of 1 µm. We used 5 ml fluorescein alcon, 10% (Alcon PHARMA, Freiburg, Germany) as an optical imaging agent. Surgery began with elevation of the apron flap, following mobilization of the larynx. The second step was to perform a pharyngotomy with cold instruments to avoid thermocoagulation damage to the mucosa. The installation of the CLE probe followed the exposure of the tumor. Subsequently, 2.5 ml fluorescein alcon 10% was injected intravenously. After around 8–10 min of examination, we applied additional 2.5 ml to increase imaging quality. We collected images of the marginal tumor region and the incision margin in the hypopharyngeal mucosa with the CLE probe. The recorded areas were marked with a suture, or a separate biopsy was performed at the precise image acquisition location. In this way, we could correlate CLE imaging with the gold standard of histopathology. The histopathological assessment followed a standard protocol with hematoxylin and eosin (H&E) staining. After completing the CLE examination, we performed the tumor resection with a macroscopic safety margin of 1 cm. Thus, our and international treatment standards were not altered or influenced in any way by the application of CLE.

### Evaluation of tissue homogeneity

**Fig. 1:**
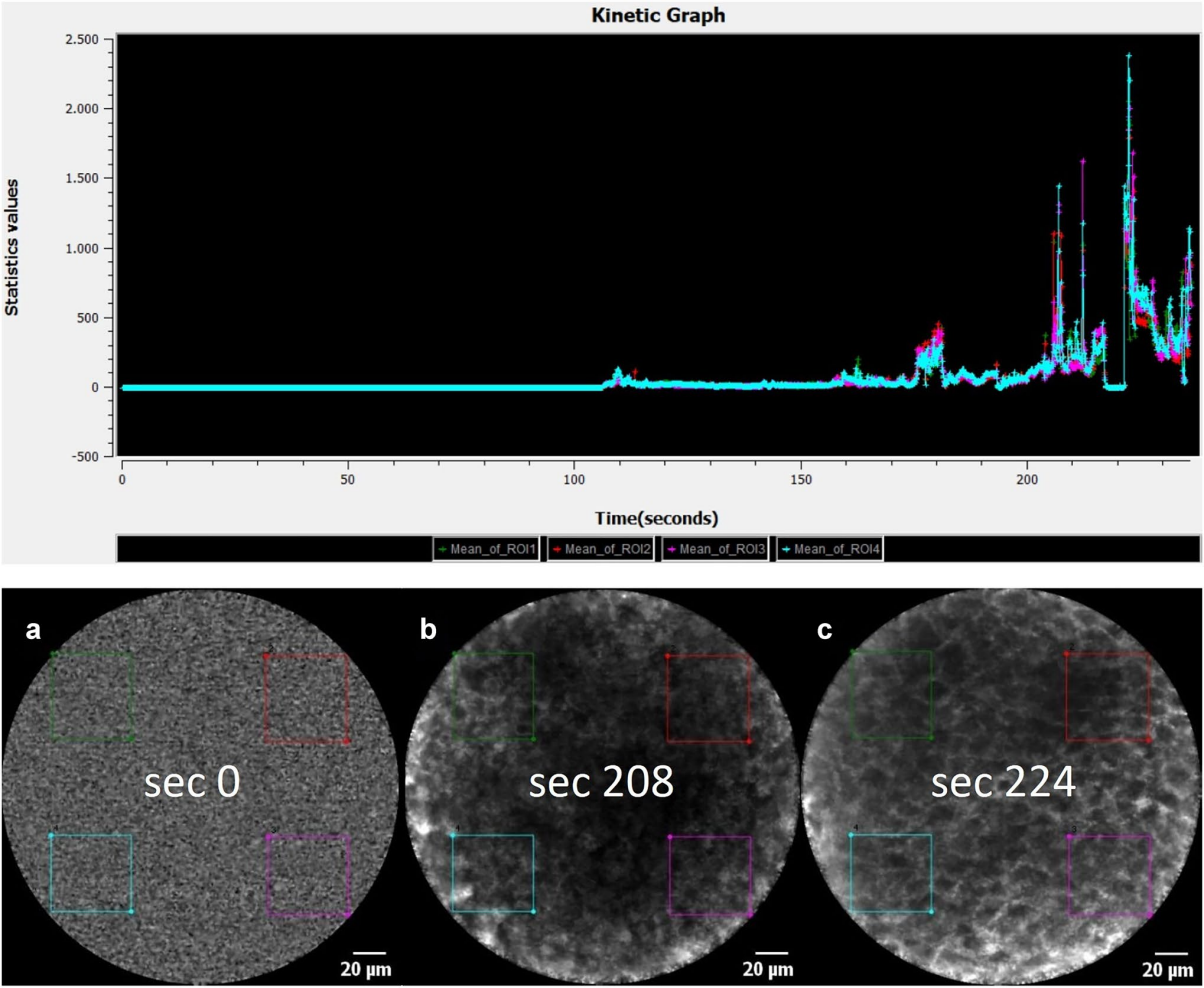
Kinetic graph shows the flooding of fluorescein after 0 (**a**), 208 (**b**), and 224 s (**c**) after i.v. application of 5 ml, fluorescein alcon, 10%. Acceptable quality is seen after 224 s after initial flooding

**Fig. 2:**
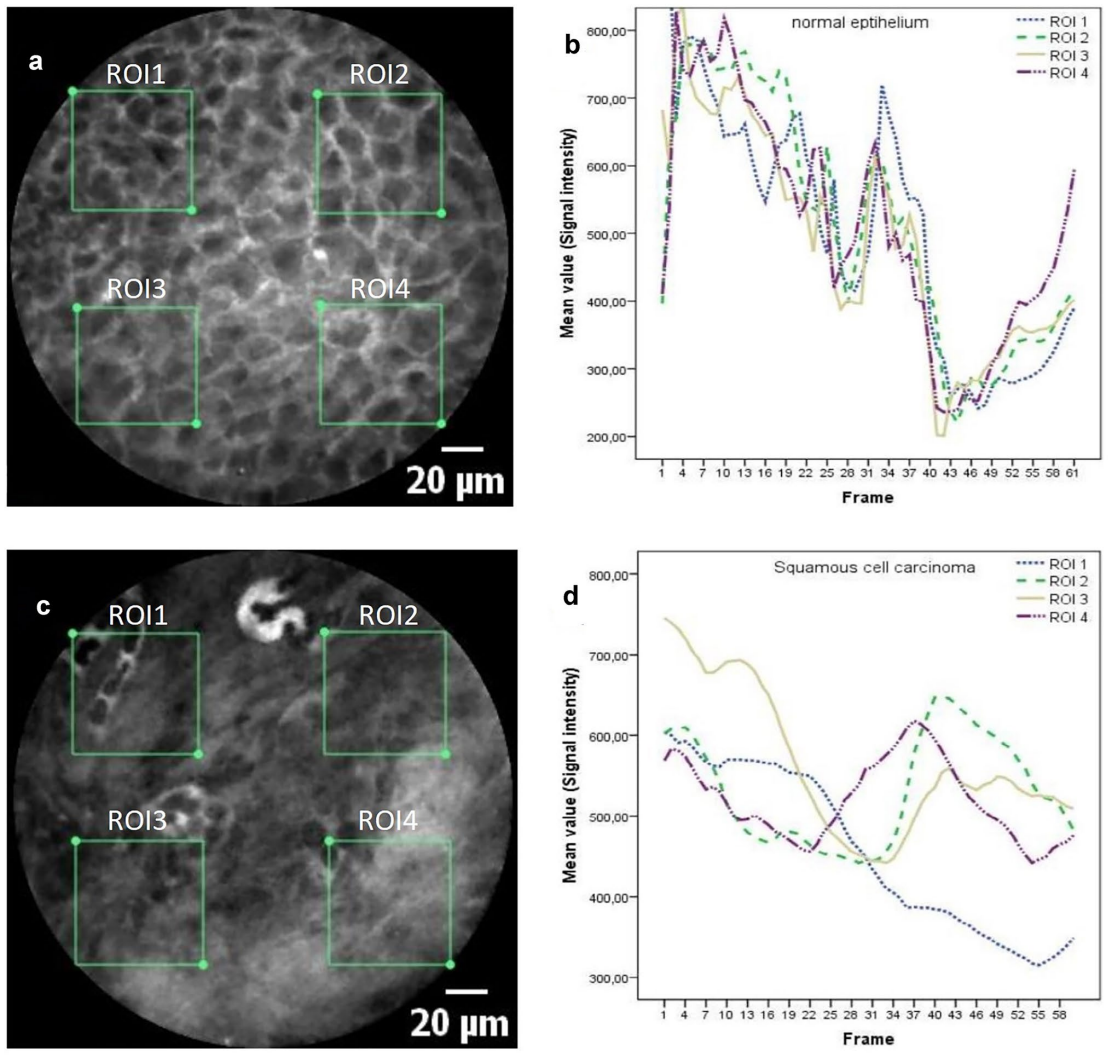
Healthy epithelium (**a**) and squamous cell carcinoma; SCC (**b**): tissue homogeneity, clear cell borders with regular, similarly sized cells with a small nucleus/cytoplasm ratio, ideally form a honeycomb pattern, are typical features of benign mucosal tissue (**a**). Malignant mucosa shows smudged intercellular spaces, atypical, tortuous vessels, and fluorescein leakage (**c**). Kinetic Graph of a 60 frame sequence (**b**, **d**). The curves almost overlap and represent the homogeneous tissue typical of healthy epithelium (**b**), whereas the curves in frames of malignant mucosa differ significantly (**d**). *ROI* region of interest

For data processing, we analyzed the sequences using Cellvizio Viewer software 1.6.2. by defining four ROIs with rectangular shapes of 45 µm × 45 µm (Figs. 1 and 2) in all four image edges. After fluorescein administration, we visually assessed tissue flooding in the region of interest (ROI) and calculated a kinetic graph of the mean histogram value (Fig. 1). After selecting the sequence with an adequate fluorescein signal, we computed a kinetic graph for each ROI. Finally, we compared four different image sectors in a sequence of 60 images over 5 s. For this, we computed the standard deviation (SD) of all four histogram values in the four ROIs to correlate the variability of signal intensity or gray-values within a sequence (Fig. 2). Furthermore, all sequences were categorized into malignant or benign by the reference standard of histopathological analysis.

In addition, we demonstrated the 60 sequences to seven independent raters in an observer-dependent assessment of tissue homogeneity, blinded to the histology, for dichotomous classification into “homogeneous” and “inhomogeneous” tissue architecture. Four raters (rater 1–4) had prior CLE experience, and three were inexperienced (rater 5–7). We defined surgeons with expertise in the technique as having performed at least 20 CLE cases.

### Statistical analysis

The two-tailed *t* test for independent samples was applied to compare each histogram's mean value and SD. For the categorical variables, we used the chi-squared test. A *P* value of less than *P* < 0.05 was considered statistically significant. The inter-rater reliability/agreement was tested using the Fleiss kappa coefficient. We interpreted κ-values according to Landis and Koch [23]. Values of κ between 0.0 and 0.20 are defined as low, between 0.21 and 0.40 as fair, between 0.41 and 0.60 as moderate, between 0.61 and 0.80 as substantial, and between 0.81 and 1.0 almost perfect. We performed statistical analysis using SPSS version 26.0 (IBM SPSS Statistics for Windows, Version 26.0. Armonk, NY, USA).

## Results

### Patient cohort

Between October 2020 and February 2021, we enrolled five patients (all male; mean age 65.4 years (SD = 11.9) to undergo in vivo CLE during planned transcervical tumor resection, concerned the hypopharynx and larynx. In one patient (20%), the tumor mass was located in the larynx. Four patients (80%) additionally presented with involvement of the hypopharyngeal mucosa. The tumor resection was performed via an open approach in each case. Microvascular defect reconstruction was necessary in three cases (60%). Regarding the tumor grading, one patient (20%) was confirmed as having an intermediate grade (G2) and four patients (80%) poor grade differentiation (G3). Patient characteristics, including stage, are presented in Table 1. In all cases, safe margin resection could be performed independent of the use of CLE. In-sano resection was defined by circular margin specimens in the intraoperative frozen section. A safety margin of > 0.5 cm was confirmed in the definitive histopathological assessment in all patients.

**Table 1:** Characteristics of patient cohort

Case No.	Age (years)	Tumor stage	Location	Grade	Surgery	CLE frames (*n*)	Recording time (seconds)	Selected sequences (*n*)
1	71	T4a	Larynx Hypopharynx	G3	Total LE, partial pharyngectomy	1468	183	14
2	56	T4a	Larynx Hypopharynx	G3	Total LE, partial pharyngectomy	2204	275	12
3	86	T4a	Larynx	G2	Total LE	2191	273	10
4	61	T2	Larynx Hypopharynx	G3	Total LE, partial pharyngectomy	3311	413	14
5	53	T3	Larynx Hypopharynx	G3	Total LE, partial pharyngectomy	2891	361	10
				Total		12,065	1505	60

*LE* laryngectomy

### Sequence selection, fluorescein flooding

With the Cellvizio Viewer 1.6.2. we assessed a kinetic graph of adequate Fluorescein flooding (Fig. 1). We recognized the initial Fluorescein enhancement after 208 s, and we obtained good quality images after 224 s. Out of 12,065 images, 60 sequences (3600 images; 33 sequences of SCC and 27 sequences of benign mucosa) were selected and evaluated as representative in acceptable quality. We matched each of these sequences (60 images, 5 s) with a corresponding sample by H&E staining to determine the diagnostic accuracy. All benign mucosal specimens were free of dysplasia or carcinoma in situ. The mean image acquisition time was 5 min (SD = 1.3) for each patient, supplemented by the assembly and disassembly of the scan unit of an additional approximately 5 min.

### Tissue homogeneity in benign and malignant mucosa

Tissue homogeneity was defined as variations in the gray-scale values between the defined quadrangular ROIs. These values were obtained as a kinetic graph (Figs. 1 and 2) and a detailed numeric list. Benign and malign frames had a mean and SD of the mean of signal intensity or gray-values of 232.1 ± 3.34 and 467.3 ± 9.72, respectively (*P* < 0.001). As a measure of variability between the four ROIs, the mean of SD in the whole 5 s/60 frame sequence was 39.1 ± 1.03 and 101.5 ± 2.6 for the healthy epithelium and SCC, respectively (*P* < 0.001).

The results of each rater are presented in Table 2. Inhomogeneous tissue architecture was observed on average in 14.8% (3.7–48.1%) of CLE sequences recorded in healthy mucosa and in 81.8% (45.4–96.9%) in CLE sequences recorded in SCC (chi-square test: *P* < 0.001). Overall sensitivity and specificity of 81.8% (95%CI: 76.3–86.2) and 86.2% (95%CI: 80.6–90.4) could be achieved, considering tissue homogeneity as the sole criterion. The sensitivity and specificity values varied from 59.1% to 96.9% and 51.8% to 100%, respectively (Table 2). The interrater reliability can be classified as moderate with a κ value of 0.53.

**Table 2:** Data analysis by the various raters

Inhomogeneity	Rater 1	Rater 2	Rater 3	Rater 4	Rater 5	Rater 6	Rater 7	All
Healthy (*n* = 27) SCC (*n* = 33)	1/27 (3.7%) 15/33 (45.4%)	13/27 (48.1%) 32/33 (96.9%)	6/27 (22.2%) 26/33 (78.7%)	1/27 (3.7%) 30/33 (90.9%)	4/27 (14.8%) 28/33 (84.8%)	1/27 (3.7%) 28/33 (84.8%)	0/27 (0%) 30/33 (90.9%)	26/189 (14.8%) 189/231 (81.8%)
Sensitivity (95%CI)	59.1% (44.4–72.3)	96.9% (84.6–99.4)	78.7% (62.2–89.3)	90.9% (76.4–96.8)	84.8% (69.0–93.3)	84.8% (69.0–93.3)	90.0% (74.3–96.5)	81.8% (76.3–86.2)
Specificity (95%CI)	93.7% (71.6–98.8)	51.8% (33.9–69.2)	77.7% (59.2–89.3)	96.3% (81.7–99.3)	85.1% (67.5–94.0)	96.3% (81.7–99.3)	100% (87.1–100)	86.2% (80.6–90.4)

All comparisons were significant in the chi-square test (*P* < 0.001)

## Discussion

This study evaluated the diagnostic value of tissue inhomogeneity of probe-based CLE in diagnosing laryngeal SCC. Carcinoma's important histological features generally include heterogeneous areas with necrosis, peritumoral inflammation, atypical mitosis, and epithelial beads [24]. Most proposed scoring systems for evaluating CLE images include tissue inhomogeneity as a conjectured correlation to the typical histological features [16–19]. An objective analysis of this feature has, however, to the best of our knowledge, not yet been performed. In the present study, we demonstrate a difference in tissue homogeneity given by the SD of the 4 ROIs in the field of view of the CLE frames with values of 39.1 ± 1.03 and 101.5 ± 2.6 for the healthy epithelium and SCC, respectively (*P* < 0.001). The identical sequences were also evaluated by seven blinded raters which identified, on average, tissue inhomogeneity in 14.8% of CLE sequences recorded in healthy mucosa and 81.8% in SCC (*P *< 0.001) and is, therefore, in line with the objective method using the kinetic graph from the Cellvizio Software. With the help of this software, the optimal point of examination could also be assessed, as tissue flooding of fluorescein is noticeable after 208 s and peaks at 224 s. This information can help us to manage and improve the time-efficiency of intraoperative examination with CLE.

We also evaluated in this study tissue homogeneity as a stand-alone feature for the diagnosis of SCC and obtained by our seven blinded raters an overall sensitivity and specificity of 81.8% and 86.2% with a moderate interrater variability of 0.53.

**Fig. 3:**
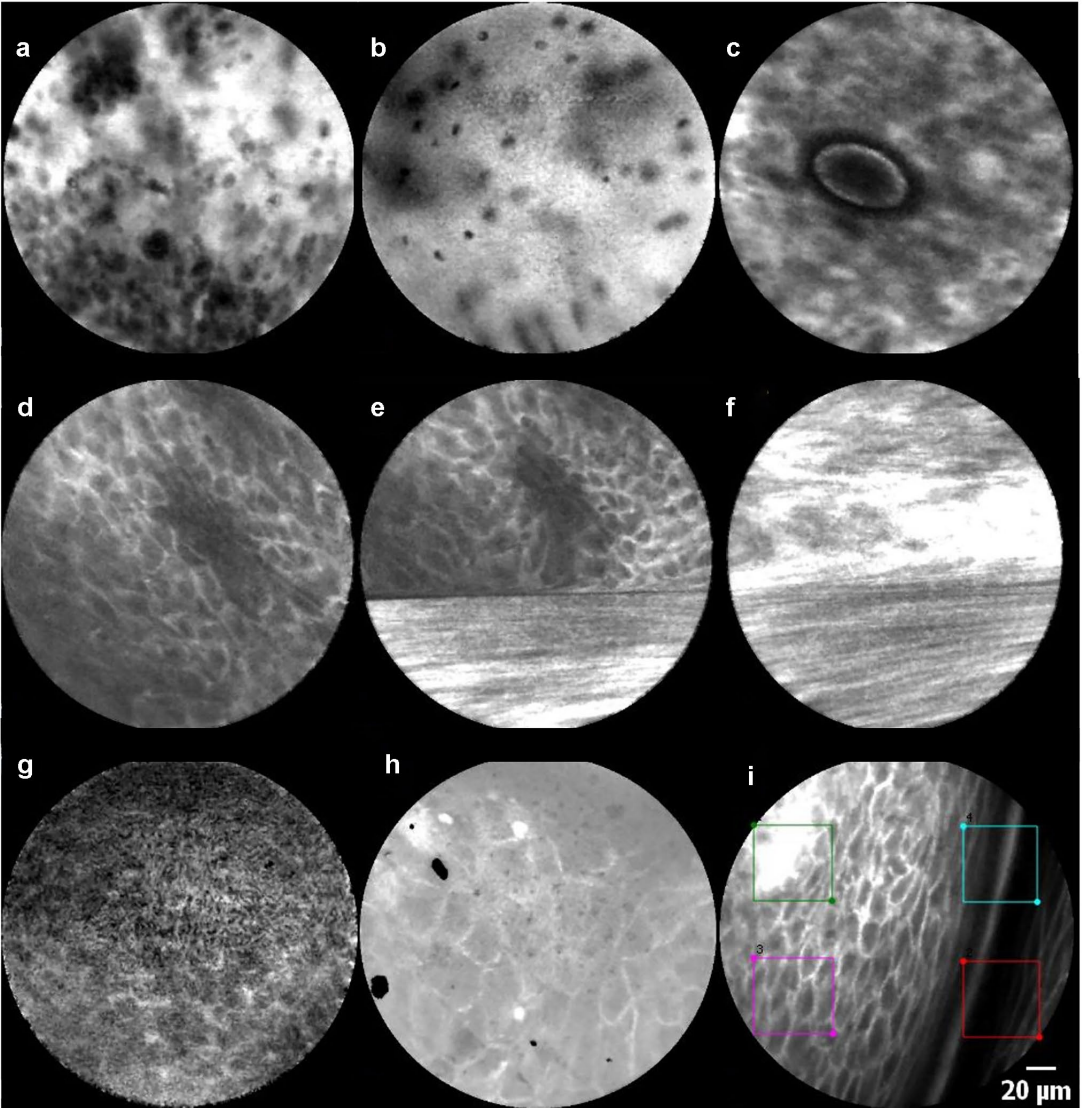
CLE image artifacts with the potential to taint image quality and cause a higher variability measured by ROI. Contamination of the probe with blood (**a**) or saliva (**b**). An air bubble due to fluid between the probe and the mucosa. Motion artifacts due to slippage of the probe (**d**–**e**); normal mucosa with the classic honeycomb pattern (**d**) and the two following frames with motion artifacts (**e** and **f**). Contact loss of the probe from the mucosa (**g**). Dirt on the lens complicates the visualization of the mucosa (**h**). Tangent position of the probe on the tissue (**i**) with an illustration of the four ROIs. The gray values of the individual ROIs differ in this frame

We found tissue homogeneity alone to be susceptible to motion artifacts (Fig. 3 e, f). As depicted in the image, motion artifacts can cover the whole frame or some sections resulting in cells appearing stretched. This issue can easily be ignored by the clinicians performing CLE in the intended setting, i.e., real-time, in-vivo for evaluation during or before oncologic surgery, as the movement of the probe relative to the underlying tissue can easily be perceived. This study shows that an automatic examination with ROI is susceptible to these movements. These translate into a more considerable SD value between the different regions of interest (Fig. 3d–f). Although a manual selection of the frames can mitigate these problems, other solutions based on artificial intelligence and deep learning algorithms that are also in development by our group are more appropriate to solve this issue [25]. The approach by Aubreville et al., based on transfer learning from intermediate endpoints within a pre-trained Inception v3 network with tailored preprocessing and fivefold cross-validation, showed an overall accuracy of 94.8% in identifying image slices tainted by motion artifacts [25]. The automatic elimination of motion artifacts can improve the classification of images for diagnosis of SCC and will be a subject of future investigations. Other artifacts such as blood, saliva, air bubbles, and debris on the surface of the probe can also taint the quality of the images and simulate regional inhomogeneity (Fig. 3a–c). AI-based approaches to eliminating these relatively common artifacts are also in development and could improve classification accuracy.

The most distinctive histological characteristic of invasive carcinoma is the penetration of the basal membrane. Due to the fixed depth of 60 μm of this technic, the basal membrane cannot be correctly evaluated. Therefore, we cannot uncritically incorporate the interpretation of CLE images from the knowledge of histopathology, and we must verify the conjectured malignancy criteria. Abbaci et al. recently showed several examples of discrepancies between CLE interpretation and final histological diagnosis by a group 3 of pathologists, such as HNSCC being interpreted as respiratory epithelium and vice versa amounting to a sensitivity and specificity of, respectively, 73.2–75% and 30–57.4%, respectively [26]. A score developed by our group to larynx and pharynx, which took tissue homogeneity, cell size, presence of cell clusters, evaluation of capillary vessels, cell size, and cell borders into consideration, showed a sensitivity and specificity of 95.1% and 86.4% for clinicians with experience in the technique, irrespective of background (pathologist or head and neck surgeon) and 86.4% and 86.1% for CLE examiners without previous experience with the method [17]. Another score developed by Oetter et al. specifically for the oral region, considering tissue homogeneity, intercellular gaps, cell morphology, fluorescence leakage, and vessel shape, showed similar sensitivity and specificity of 95.3% and 88.9%, respectively [16]. Although these diagnostic metrics do not enable CLE to be a substitute for histopathology, if nothing else, because only a statement regarding presence or absence of malignancy is possible at the moment, they do come close to the values of intraoperative frozen sections, which show accuracy levels of 98.6–88.2% [27, 28].

In a previous study about the diagnostic value of intraepithelial capillary loops and atypical vessels in SCC diagnosis, we demonstrated that a cutoff value for vessel diameter of 30 μm enables the diagnosis of SCC with a sensitivity and specificity of, respectively, 90.6% and 71.3% [29]. However, while both criteria, tissue homogeneity and vessel diameter, as stand-alone diagnostic features, provide good sensitivity and specificity values, its diagnostic metrics are still inferior to diagnostic scores that integrate both features [17].

Histological examination is performed with a vertical slice through the different tissue layers. In contrast, probe-based CLE provides a tangential depiction of the epithelium at a fixed depth with a defined limited field of view [14, 17, 22]. With its 5–10 cell layers, the laryngeal epithelium has an average of 150 μm thickness [30, 31]. Arens et al. showed in a total of 206 vocal fold lesions that a progressive thickening of vocal cord epithelium over the different grades of dysplasia occurs with early invasive carcinoma showing an average epithelial thickness of 974 μm with a minimum of 800 μm [30]. However, the fixed depth of examination of 60 μm is not sufficient to evaluate the basal membrane. Therefore, this determinant in the histopathological differentiation between carcinoma in situ and invasive carcinoma cannot be used. Currently, there is no data comparing carcinoma in situ with invasive carcinoma using tissue homogeneity and capillary structure criteria [32]. However, this should be part of future research.

The rationale for non-invasive in vivo diagnostics is to define and control the resection margins precisely. The probe enables scanning the entire surgical field in just a few minutes. It allows the surgeon to get an overview of the extent of resection even with three-dimensional defects. The CLE may increase the probability of achieving an in-sano resection at the "first strike" and decrease the risk for re-resection, as the positive resection margin appeared to be an independent adverse prognostic factor [33]. In addition, the CLE can help take frozen sections in a more targeted manner to ensure the safety of the in-sano resection in communication with the pathologist. Thus, CLE offers the possibility to keep the resection volume as low as possible and, at the same time to realize a sufficient assessment of the entire resection margin. Furthermore, the clear differentiation between benign mucosal changes, normal mucosa, and malignant changes is essential in early tumor diagnostic. In addition, CLE significantly helps to improve tumor follow-up monitoring for possible local recurrence and reduces the risk caused by unnecessary biopsies.

Other optical techniques, such as narrow-band imaging (NBI), have shown promising results in tumor diagnosis in the upper aerodigestive tract with an overall sensitivity and specificity of 89% and 96% [34]. This technique differs fundamentally from CLE. Due to its natural contrasted evaluation of vascular patterns in large mucosal areas [35] it can be considered a horizontal method to easily detect suspicious mucosal regions compared to white light endoscopy [36]. The magnification provided by NBI does not allow for the depiction of the cellular level. In addition to visualizing mucosal capillaries and atypical, aberrant vessels, CLE can also visualize superficial epithelial cells' contour and tissue architecture with up to ×1000 magnification, resulting in an additional dimension for healthy mucosa and squamous cell carcinoma differentiation contrast to NBI. CLE, however, only enables the examination of a small section of a few millimeters at a time (2.6 mm diameter in this study) and requires the application of a fluorescent dye. CLE with simultaneous NBI-endoscopy could potentially enable a more accurate diagnostic. However, there are no studies at the moment that show the benefit of the combined application of both techniques.

## Conclusions

The precise analysis of tissue homogeneity is a promising approach to improving CLE’s diagnostic value to differentiate malignant squamous epithelial lesions and benign mucosa. Malignant lesions often have an inhomogeneous appearance on CLE examination and can be identified based on this feature with 81.8% and 86.2% sensitivity and specificity. Furthermore, the in-vivo, real-time examination by the clinician performing the technique during oncological surgery mitigates some weaknesses of the automatic analysis of tissue homogeneity in whole sequences, such as motion artifacts, saliva, blood, and debris, which can be directly identified as the cause for an apparent inhomogeneity. In addition, CLE enables the depiction of other criteria, which, when taken together in consideration, can provide a higher diagnostic value as a single-criterion examination considering only tissue homogeneity.
